# Beta-Blockers and Oxidative Stress in Patients with Heart Failure

**DOI:** 10.3390/ph4081088

**Published:** 2011-08-05

**Authors:** Kazufumi Nakamura, Masato Murakami, Daiji Miura, Kei Yunoki, Kenki Enko, Masamichi Tanaka, Yukihiro Saito, Nobuhiro Nishii, Toru Miyoshi, Masashi Yoshida, Hiroki Oe, Norihisa Toh, Satoshi Nagase, Kunihisa Kohno, Hiroshi Morita, Hiromi Matsubara, Kengo F Kusano, Tohru Ohe, Hiroshi Ito

**Affiliations:** 1 Department of Cardiovascular Medicine, Okayama University Graduate School of Medicine, Dentistry and Pharmaceutical Sciences, 2-5-1 Shikata-cho, Kita-ku, Okayama 700-8558, Japan; 2 Department of Cardiovascular Therapeutics, Okayama University Graduate School of Medicine, Dentistry and Pharmaceutical Sciences, 2-5-1 Shikata-cho, Kita-ku, Okayama 700-8558, Japan; 3 Division of Cardiology, National Hospital Organization Okayama Medical Center, 1711-1 Tamasu, Kita-ku, Okayama 701-1192, Japan

**Keywords:** beta-blocker, oxidative stress, heart failure

## Abstract

Oxidative stress has been implicated in the pathogenesis of heart failure. Reactive oxygen species (ROS) are produced in the failing myocardium, and ROS cause hypertrophy, apoptosis/cell death and intracellular Ca^2+^ overload in cardiac myocytes. ROS also cause damage to lipid cell membranes in the process of lipid peroxidation. In this process, several aldehydes, including 4-hydroxy-2-nonenal (HNE), are generated and the amount of HNE is increased in the human failing myocardium. HNE exacerbates the formation of ROS, especially H_2_O_2_ and ·OH, in cardiomyocytes and subsequently ROS cause intracellular Ca^2+^ overload. Treatment with beta-blockers such as metoprolol, carvedilol and bisoprolol reduces the levels of oxidative stress, together with amelioration of heart failure. This reduction could be caused by several possible mechanisms. First, the beta-blocking effect is important, because catecholamines such as isoproterenol and norepinephrine induce oxidative stress in the myocardium. Second, anti-ischemic effects and negative chronotropic effects are also important. Furthermore, direct antioxidative effects of carvedilol contribute to the reduction of oxidative stress. Carvedilol inhibited HNE-induced intracellular Ca^2+^ overload. Beta-blocker therapy is a useful antioxidative therapy in patients with heart failure.

## 1. Introduction

Reactive oxygen species (ROS) induce ‘oxidative stress’ unless the prepared antioxidant mechanisms compensate for the ROS load [1]. Oxidative stress has been implicated in the pathogenesis of cardiovascular disease [2,3], including heart failure [4,5]. Increased oxidative stress results from an imbalance between ROS load and antioxidative mechanisms. Catecholamines [6-8], angiotensin II [9-11], tumor necrosis factor-alpha (TNF-alpha) [9], tachycardia and ischemia [12] induce generation of ROS in the myocardium. Activities of some antioxidant enzymes such as paraoxonase-1 (PON-1) in serum and manganese superoxide dismutase (MnSOD) in the myocardium, if not all enzymes, are diminished in patients with heart failure [7,13-15]. Thus, oxidative stress levels are elevated in the failing myocardium [6,16-18], and ROS causes hypertrophy, apoptosis/cell death and intracellular Ca^2+^ overload in cardiac myocytes [7,9,10,19-21]. In this review, we will focus on the involvement of oxidative stress in patients with heart failure and its reduction by beta-blocker therapy.

## 2. Involvement of Oxidative Stress in Heart Failure

In 1991, Belch *et al*. reported that concentrations of a plasma lipid peroxide, malondialdehyde, were significantly higher in the patients with congestive heart failure than in controls [4]. There was a significant negative correlation between malondialdehyde and left ventricular ejection fraction. Mallat *et al*. reported that pericardial levels of 8-iso-prostaglandin F_2a_ (8-iso-PGF_2a_), a specific nonenzymatic peroxidation product of arachidonic acid, increase with increase in the functional severity of heart failure and are associated with ventricular dilatation [22].

**Figure 1:**
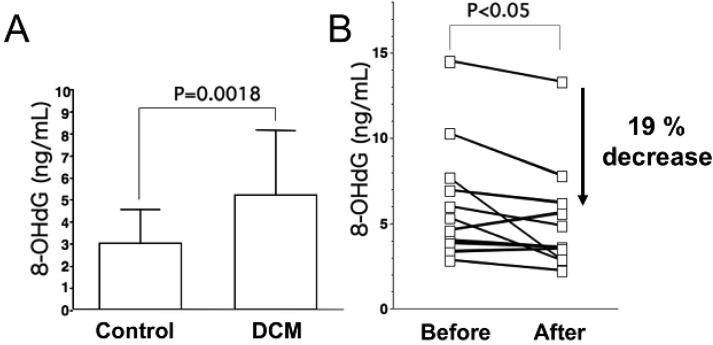

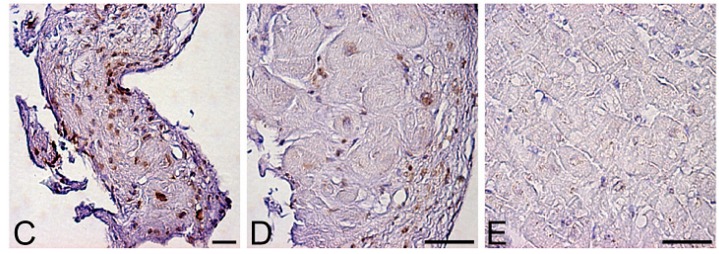
Elevated levels of oxidative DNA damage in serum and myocardium of patients with heart failure [18].

As for oxidative stress markers other than lipid peroxides, levels of 8-hydroxy-2-deoxyguanosine (8-OHdG), a marker of oxidative DNA damage, are elevated in serum and urine of patients with heart failure, and urinary 8-OHdG reflects the clinical severity of CHF on the basis of symptomatic status and cardiac dysfunction (Figure 1A) [18,23]. Results of these studies indicate that oxidative stress is involved in severity of heart failure.

**Figure 2:**
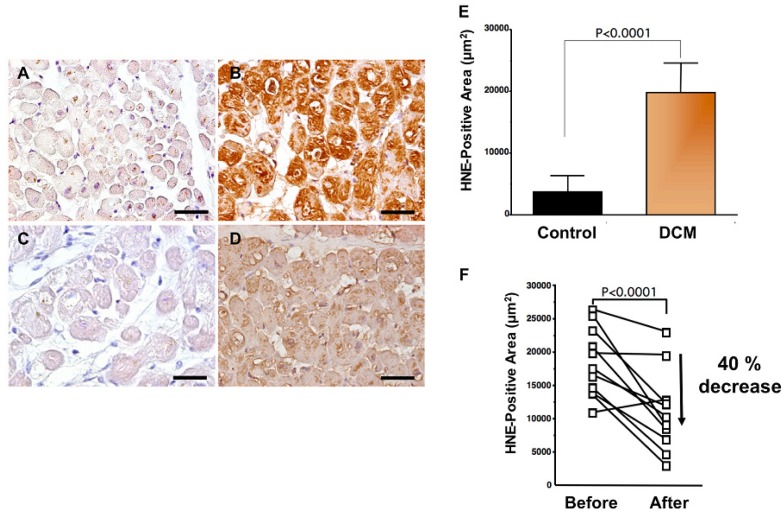
Carvedilol decreases elevated oxidative stress in the human failing myocardium [6].

Levels of oxidative stress are also elevated in the myocardium of patients with heart failure. ROS cause damage to lipid cell membranes in the process of lipid peroxidation. In this process, several aldehydes, including 4-hydroxy-2-nonenal (HNE), are generated and the amount of HNE is increased in the human failing myocardium [Figures 2(B,E)] [6,17]. The presence of 8-OHdG has also been detected in nuclei of cardiac myocytes in patients with dilated cardiomyopathy (DCM) [Figures 1(C,D) [18]. Therefore, levels of oxidative stress are elevated in both body fluid including serum, urine and pericardial effusion and myocardium of patients with heart failure.

HNE is recognized not only as a reliable marker of oxidative stress but also as a toxic aldehyde to many types of cells [1,24-26]. HNE exhibits cytopathological effects, such as enzyme inhibition and inhibition of DNA, RNA and protein synthesis. HNE exacerbates heart failure. Administration of HNE was found to cause contractile failure and to elicit proarrhythmic effects in hearts [27,28]. HNE also has pro-oxidant properties.

**Figure 3:**
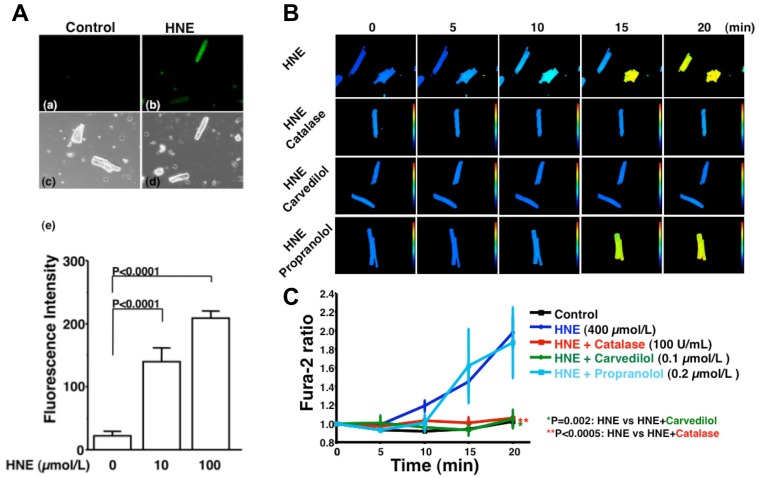
4-Hydroxy-2-nonenal (HNE) induces calcium overload *via* the generation of reactive oxygen species in isolated rat cardiac myocytes [21].

**Figure 4:**
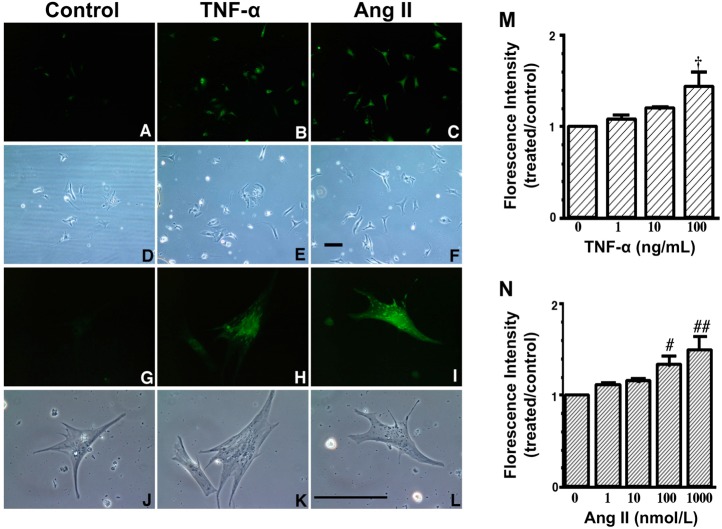
Generation of ROS in neonatal rat cardiac myocytes induced by tumor necrosis factor-α (TNF-α) and angiotensin II (Ang II) [9].

**Figure 5:**
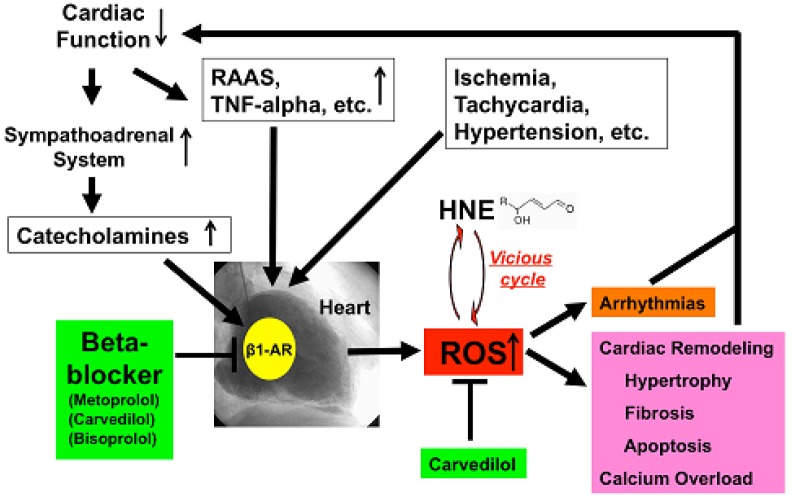
Termination of vicious cycles of oxidative stress and depressed cardiac function by treatment with a beta-blocker.

HNE can markedly induce intracellular production of ROS in cultured rat hepatocytes and human neuroblastoma cells [26,29,30]. HNE also exacerbates the formation of ROS, especially H_2_O_2_ and ·OH, in cardiomyocytes and subsequently ROS cause intracellular Ca^2+^ overload (Figure 3) [21]. Therefore, HNE exacerbates heart failure by increasing oxidative stress in the myocardium. Angiotensin II [9-11], catecholamines [6-8], tumor necrosis factor-alpha (TNF-alpha) [9] and ischemia [12] induce generation of ROS in the myocardium (Figure 4). ROS cause HNE formation in the process of lipid peroxidation. HNE inversely induces generation of ROS. In this way, a vicious cycle of oxidative stress is formed in the failing myocardium (Figure 5). Terminating this vicious cycle may be important to reduce oxidative stress in the failing myocardium.

Different oxidative stress levels stimulate cellular proliferation, trigger apoptosis or produce necrosis in various cells [1,31]. Experimental studies have shown that ROS can exert a graded effect on the cardiac myocyte phenotype [7,32]. Lower levels of oxidative stress stimulate hypertrophy and higher levels of oxidative stress induce apoptosis [7,32]. Higher rates of ROS production contribute to the transition from compensatory left-ventricular hypertrophy to heart failure [33,34]. We previously reported that oxidative stress was elevated in myocardia of patients with hypertrophic cardiomyopathy and that the levels were correlated with left ventricular dilatation and systolic dysfunction [17]. Thus, graded effects of ROS on the myocyte phenotype may be present in clinical settings. Further studies are needed to clarify this point.

## 3. Reduction of Oxidative Stress by Beta-blocker Therapy

### 3.1. Reduction Effect of a Beta-blocker on Oxidative Stress in Patients with Heart Failure

Results of many clinical trials, including the US Carvedilol Heart Failure Study, CIBIS-II and III, MERIT-HF, CARMEN, MUCHA, COMET, COPERNICS and REVERT have supported the efficacy of three beta-blockers (carvedilol, metoprolol and bisoprolol) in the treatment of heart failure. These studies have revealed that both ß_1_ adrenoreceptor blockers (metoprolol and bisoprolol) and a nonselective ß_1_ and ß_2_ adrenoreceptor blocker (carvedilol) ameliorate cardiac function and mortality in patients with heart failure. One of the mechanisms is thought to be reduction effect of a beta-blocker on oxidative stress. Kukin *et al.* reported that both metoprolol, a β_1_-selective blocker, and carvedilol, an α and β blocker with antioxidant activity, reduced plasma lipid peroxidation in patients with heart failure, together with amelioration of heart failure [35]. Chin *et al.* reported that β**-**blockers (bisoprolol and carvedilol), reduced the levels of serum lipid hydroperoxides in patients with CHF [36]. The serum levels of 8-OHdG in patients with DCM significantly decreased by 19% (Figure 1B) [18]. Thus, treatment with a beta-blocker can reduce systemic levels of oxidative stress, along with amelioration of heart failure.

A beta-blocker decreases elevated oxidative stress not only in serum or plasma but also in the human failing myocardium [6]. Endomyocardial biopsy samples from 11 patients with DCM were examined before and after treatment (mean, 9 ± 4 months) with carvedilol (5 to 30 mg/day; mean dosage, 22 ± 8 mg/day). After treatment with carvedilol, myocardial HNE-modified protein levels decreased by 40% along with amelioration of cardiac function (Figures 2B,D,F) [6]. Since HNE is a cytotoxic product, the reducing effects of carvedilol may play a critical role in amelioration of heart failure.

### 3.2. Mechanism of Reduction of Oxidative Stress by Beta-blocker Therapy

This reduction can be caused by several possible mechanisms.

#### 3.2.1. Class Effect of Beta-Blockers

a. **ß_1_-blocking effect.** Isoproterenol induces lipid peroxidation [8]. Apoptosis stimulated by ß_1_-adrenergic receptors (ß_1_-AR) may also be mediated by ROS production [7]. Therefore, ß_1_-adrenergic receptor blockers are useful for catecholamine-induced oxidative stress.
b. **Anti-ischemic properties**. Anti-ischemic properties may also be important since ischemia has been shown to increase HNE formation in the heart [12].
c. **Negative chronotropic effect.** Tachycardia induces ROS generation in mitochondria of the myocardium [16]. Therefore, a negative chronotropic effect may be important.
d. **Anti-hypertensive effect.** Mechanical strain in cardiac myocytes increases ROS production [37,38]. Lowering blood pressure by a beta-blocker reduces after-load of the heart and finally may reduce ROS production.

#### 3.2.2. Specific Effect of Carvedilol

e. **Direct antioxidative property of carvedilol.** The direct antioxidative property of carvedilol may contribute to the reduction of oxidative stress. Carvedilol inhibited Fe^2+^-initiated lipid peroxidation *in vitro,* but propranolol did not [39]. The mechanism of inhibition is via scavenging free radicals [39]. Carvedilol prevented hydroxyl radical-induced cardiac contractile dysfunction in human myocardial tissue, but metoprolol did not [40]. These results suggest the possible importance of the use of carvedilol.
f. **Inhibitory effects of carvedilol on ROS generation by leukocytes.** Carvedilol inhibits ROS generation by leukocytes [41].
g. α**-blocking effect of carvedilol.** Xiao *et al.* reported that ventricular myocytes express components of an NAD(P)H oxidase that appear to be involved in α_1_-AR-stimulated hypertrophic signaling *via* ROS-mediated activation of Ras-MEK1/2-ERK1/2. Therefore, the α-blocking effect of carvedilol may reduce oxidative stress [42].

#### 3.2.3. Future Perspective of Mechanism Analysis

Emerging studies have revealed that β adrenergic receptor polymorphism may have an impact on response of β blocker treatment in patients with heart failure [43,44]. For example, there is a polymorphism at amino acid residue 389 (Arg/Gly) in human adrenergic β_1_-receptor gene (ADRB2). Mialet Perez *et al.* reported that homozygosity for Arg389 was associated with improvement in ventricular function during carvedilol treatment in 224 patients with heart failure [43]. A large clinical trial and further experimental examinations are needed to clarify this response. Determination of whether antioxidative effects of β blockers are different between Arg389 and Gly389 may be important.

Endothelial function is impaired in patients with heart failure [45] and is also related to elevated oxidative stress [46]. The third-generation β blocker nebivolol improves left ventricular dysfunction and have an effect on NO-mediated endothelial function in mice with extensive anterior myocardial infarction [47]. Nebivolol inhibits superoxide formation by vascular NADPH oxidase activation in angiotensin II-treated rats [48] and by vascular NOS III uncoupling in Watanabe heritable hyperlipidemic rabbits [49] and prevents endothelial dysfunction. Further studies are needed to clarify the beneficial effects of nebivolol in clinical settings [50,51].

## 4. Conclusions

### Beta-blocker as a Possible Antioxidant in Treatment for Heart Failure

Depressed cardiac function causes activation of the sympathoadrenal system (ANS) and rennin-angiotensin-aldosterone system (RAAS) and elaboration of cytokines such as TNF-alpha [52]. All of these ischemia, tachycardia or hypertension increased ROS generation in the human failing myocardium (Figure 5). Catecholamines generate ROS via β_1_-AR, and beta-blockers, including metoprolol, carvedilol and bisoprolol, can therefore decrease ROS generation in the heart. Furthermore, carvedilol can scavenge redundant ROS and can terminate the vicious cycle such as oxidative stress caused by the cytotoxic aldehyde, HNE. ROS cause remodeling, hypertrophy, fibrosis, apoptosis and calcium overload and induce arrhythmia [53], which in turn depresses cardiac function. Beta-blockers such as metoprolol, bisoprolol and carvedilol can stop those vicious cycles in patients with heart failure via indirect or direct anti-oxidative properties.

Antioxidants to ameliorate cardiovascular diseases including heart failure have not generally yielded favorable results [54]. Furthermore, small or adequate degrees of stimulation by ROS are physiological and are needed in the bio-defense system or anti-tumorigenesis [54,55]. Thus, total blocking of oxidative stress in patients with heart failure is not needed or it may be harmful. In our study, carvedilol decreased the serum levels of 8-OHdG by 19% (Figure 1B) [18] and myocardial HNE-modified protein levels by 40% along with amelioration of cardiac function in patients with heart failure [Figures 2B,D,F] [6]. Since the therapy is safe and reduces patient mortality, the antioxidative properties of a beta-blocker may be sufficient to treat patients with heart failure. Further investigations are needed to clarify this point. In conclusion, beta-blocker therapy is an antioxidative therapy that is useful and safe in patients with heart failure.

## Figures and Tables

**A**. Serum levels of 8-hydroxy-2-deoxyguanosine (8-OHdG), a marker of oxidative DNA damage, in patients with dilated cardiomyopathy (DCM). DCM patients had significantly elevated serum levels of 8-OHdG compared with those in control subjects. Data are given as mean ± SD. **B**. Decrease in serum 8-OHdG levels due to treatment with carvedilol. Serum 8-OHdG levels decreased by 19% during treatment with carvedilol. C, D and E, immunohistochemical examination of 8-OHdG in myocardial biopsy samples. **C**. Representative immunohistochemical staining (brown) of a myocardial biopsy sample from a patient with DCM in a low-power field. Positive staining (brown) for 8-OHdG was distinct in the nuclei of cardiac myocytes. **D**. Representative staining of a myocardial biopsy sample from a patient with DCM in a high-power field. **E**. Representative staining in a myocardial biopsy sample from a control subject. Bar = 50 μm.

**A to D**. Immunohistochemical examination of HNE-modified protein in myocardial biopsy samples. **A**. Representative immunohistochemical staining in a myocardial biopsy sample from a control subject. There is no positive staining in cardiac myocytes; **B**. Representative staining (brown) in a myocardial biopsy sample from a dilated cardiomyopathy (DCM) patient before carvedilol treatment. Positive staining (brown) for HNE-modified protein was distinct in the cytosol of cardiac myocytes; **C**. Negative control sections incubated without a primary antibody; **D**. Representative staining in a myocardial biopsy sample from the same DCM patient 9 months after the beginning of carvedilol treatment. HNE-positive area is diminished. Bar = 50 μm; **E**. Expression levels of HNE-modified protein (HNE-positive area) in patients with DCM. DCM patients had significantly elevated myocardium levels of HNE-modified protein compared with those in control subjects. Data are given as mean ± SD; **F**. Decrease in HNE-positive area attributable to treatment with carvedilol. During treatment with carvedilol, the HNE-positive area decreased by 40%.

**A**. Generation of ROS induced by HNE exposure. Analysis of dichlorofluorescein (DCF) fluorescence. DCF is a non-specific detector of the production of intracellular ROS such as H_2_O_2_, ·OH and hydroperoxides (ROOH). (a–d), These are representative living cardiac myocytes observed by fluorescence microscopy (a and b) and phase contrast microscopy (c and d). Isolated rat cardiac myocytes on glass-base dishes were treated with a diluent (control) (a and c), HNE (100 μmol/L) (b and d). Treatment time was 10 minutes. (e), DCF fluorescence intensity per cell (n = 13 cells). Treatment with HNE (10 to 100 μmol/L) for ten minutes also induced the production of ROS (H_2_O_2_, OH or ROOH) in isolated rat ventricular myocytes in a dose-dependent manner, as assessed by DCF fluorescence. Each point is the mean ± SE. B and C. Inhibitory effects of antioxidants on the increase in fura-2 ratio due to HNE exposure; **B**. These are representative fura-2 ratio images in living cardiac myocytes observed by fluorescence microscopy. Isolated rat cardiac myocytes on glass-base dishes were treated with HNE (400 μmol/L), HNE and catalase (100 U/mL), HNE and carvedilol (0.1 μmol/L), or HNE and propranolol (0.2 μmol/L). Treatment time was 20 minutes; **C**. Antioxidants, catalase (n = 6) or carvedilol (n = 6), significantly inhibited the increase in fura-2 ratio. Propranolol did not inhibit the increase in fura-2 ratio (n = 7). Each point is the mean ± SE. HNE (400 μmol/L) exposure increased [Ca^2+^]_i_ of isolated cardiac myocytes as assessed by fura-2 ratio in a time-dependent manner. Catalase (100 U/mL), an antioxidative enzyme, significantly attenuated the increase in [Ca^2+^]_i_ induced by HNE. Furthermore, carvedilol (0.1 μmol/L), a beta-blocker with potent antioxidant activity, significantly attenuated the increase in [Ca^2+^]_i_ induced by HNE, but propranolol (0.2 μmol/L) had no effect on [Ca^2+^]_i_ increase. These results indicate that ROS mediate HNE-induced intracellular Ca^2+^ overload.

**A to L**. Increase in fluorescence with 2′,7′- dichlorofluorescin diacetate (DCFH-DA) due to TNF-α and Ang II exposure. These are representative living cardiac myocytes observed by fluorescence microscopy (A–C, G–I) and phase contrast microscopy (D–F, J–L). On culture day 4, cultured cardiac myocytes on glass cover slips were treated with a diluent (control) (left: A, D, G and J), TNF-α (10 ng/mL) (middle: B, E, H and K) or Ang II (100 nmol/L) (right: C, F, I and L) and with DCFH-DA (5 μmol/L). Treatment time was 1 hour. Upper two lanes (A–F) at low magnification and lower two lanes (G–L) at high magnification. Bar = 100 μm. M and N, Dose-dependent increase in fluorescence due to TNF-α and Ang II exposure. On culture day 4, cultured cardiac myocytes were treated with TNF-α (1 to 100 ng/mL), Ang II (1 to 1000 nmol/L) or a diluent without TNF-α and Ang II (control) and simultaneously with 2′,7′- dichlorofluorescin diacetate (DCFH-DA) (1 μmol/L). After 1 hour of incubation, cells were collected and fluorescence intensity per culture well was measured. Each point is the mean ± SE (n = 5 experiments). In each experiment, a treated-to-control ratio was calculated from the fluorescence intensity of five experiments. † *P* < 0.005, # *P* < 0.01, ## *P* < 0.0005 vs control cultures treated with diluent only. These data indicate that both TNF-α and Ang II generate ROS in cardiac myocytes.
